# Triglyceride-rich lipoprotein, remnant cholesterol, and apolipoproteins CII, CIII, and E in patients with schizophrenia

**DOI:** 10.1016/j.jlr.2024.100577

**Published:** 2024-06-13

**Authors:** Jeffrey Wang, Maaike Kockx, Magdalena Bolek, Tim Lambert, David Sullivan, Vincent Chow, Leonard Kritharides

**Affiliations:** 1 Atherosclerosis Laboratory, ANZAC Research Institute, Concord Repatriation General Hospital and the University of Sydney, Sydney, Australia; 2 Concord Clinical School, Faculty of Medicine and Health, University of Sydney, Sydney, Australia; 3 Collaborative Centre for Cardiometabolic Health, Charles Perkins Centre, University of Sydney, Sydney, Australia; 4 Department of Chemical Pathology, Royal Prince Alfred Hospital, Sydney, Australia; 5 Central Clinical Medical School, Faculty of Medicine and Health, University of Sydney, Sydney, Australia; 6 Department of Cardiology, Concord Repatriation General Hospital, Sydney, Australia

**Keywords:** schizophrenia, triglycerides, remnant cholesterol, triglyceride-rich lipoproteins, apolipoproteins, angiopoietin-like proteins

## Abstract

Patients with schizophrenia show a disproportionally increased risk of cardiovascular disease. Hypertriglyceridemia is prevalent in this population; however, how this relates to levels of remnant cholesterol, triglyceride (TG)-rich lipoprotein (TRL) particle size and composition, TG turnover, and apolipoprotein (apo) and angiopoietin-like protein (ANGPTL) concentrations is unknown. Fasting levels of cholesterol (total [TC], LDL-C, HDL-C, non-HDL-C and remnant cholesterol) and TG were determined in 110 patients diagnosed with schizophrenia, and 46 healthy controls. TRL particle size, concentration and composition, and β-hydroxybutyrate (TG turnover marker) were assessed by NMR. Levels of apoCII, apoCIII, apoE, ANGPTL3, ANGPTL4, and ANGPTL8 were measured by ELISA, and apoCII, apoCIII and apoE were further evaluated in HDL and non-HDL fractions. Patients with schizophrenia had significantly elevated TG, TG:apoB ratio, non-HDL-C, remnant cholesterol, non-HDL-apoCII and non-HDL-apoCIII, and HDL-apoE (all *P* < 0.05), lower HDL-C and apoA-I (all *P* < 0.001), and comparable apoB, TC, TC:apoB ratio, LDL-C, β-hydroxybutyrate, ANGPTL3, ANGPTL4 and ANGPTL8 to healthy controls. Patients had a 12.0- and 2.5-fold increase in the concentration of large and medium TRL particles respectively, but similar cholesterol:TG ratio within each particle. Plasma TG, remnant cholesterol, and large and medium TRL particle concentrations correlated strongly with apoCII, apoCIII, and apoE in the non-HDL fraction, and with apoCIII and apoE in the HDL fraction in patients with schizophrenia. Differences in TG, HDL-C, TRL particle concentrations, apoCIII, and apoE persisted after adjustment for conventional risk factors. These results are consistent with impaired TRL lipolysis and clearance in patients with schizophrenia which may be responsive to targeting apoCIII.

Patients with schizophrenia (SZ) experience disproportionate rates of medical morbidity and mortality and die on average 25 years earlier compared to the general population (1). Most excess mortality is due to cardiovascular disease (CVD). On average, one in three illness-related deaths are due to CVD, and over half of these are ischemic heart disease-related (2). Obesity, diabetes and dyslipidemia are more prevalent in patients with SZ. Furthermore, they often exhibit features typical of the metabolic syndrome with elevated body mass index (BMI), fasting blood glucose, blood pressure, triglycerides (TGs), and decreased high-density lipoprotein cholesterol (HDL-C), all of which contribute to increased cardiovascular morbidity and mortality (3). Clinical observations have shown that in part these modifiable risk factors are attributable to an unhealthy lifestyle including poor diet, physical inactivity and smoking and substance use, as well as the use of antipsychotic and orexigenic psychotropic medications (4, 5). Despite this, cardiovascular risk factors remain undertreated in patients with SZ.

Elevated plasma TG is recognized as an established risk factor for incident CVD independent of elevated low-density lipoprotein cholesterol (LDL-C) levels (6). TGs are predominantly transported in the bloodstream as TG-rich lipoproteins (TRLs), and these include chylomicrons that are produced from the intestine, very low-density lipoproteins (VLDLs) that are secreted from the liver, and intermediate-density lipoproteins (IDLs) that are derived from VLDLs. In addition to TGs, TRLs also transport cholesterol and phospholipids and carry structural and functional proteins that regulate their composition and biological fate (7). Lipoprotein lipase (LPL) is a key mediator of TG hydrolysis on TRLs and leads to the formation of cholesterol-rich TRL remnants, which are cleared via hepatic uptake. In contrast to chylomicrons and VLDLs, remnant particles are small and have been demonstrated to enter and be retained in the arterial wall, and therefore are directly atherogenic (8). Deficiency in LPL activity and/or decreased hepatic clearance lead to increased TRL particle numbers and accumulation of remnant particles, and are associated with increased risk of CVD (7). Furthermore, elevated remnant cholesterol levels are causally linked to low-grade inflammation and atherosclerosis (9, 10). In addition, TG hydrolysis leads to the generation of free fatty acids that undergo β-oxidation and ketogenesis in the liver yielding the ketone body β-hydroxybutyrate, which has been recently demonstrated to be a marker of TG turnover associated with increased risk of cardiovascular mortality (11).

LPL activity is regulated by the actions of several regulatory proteins, which have been linked to hypertriglyceridemia, remnant generation, and CVD risk. Apolipoprotein C-II (apoCII) is an important co-factor that is required for the activation of LPL activity. Consistent with this, apoCII deficiency in patients and rodent models is associated with severe hypertriglyceridemia with markedly elevated TG and chylomicron levels (12, 13). In contrast, apoCIII and apoE are thought to inhibit LPL activity (14, 15, 16). Previous studies have shown that these apolipoproteins may also modulate TG and remnant levels by regulating TRL uptake and clearance by the liver (17, 18). Additionally, circulating factors such as the angiopoietin-like proteins 3, 4 and 8 (ANGPTL3, ANGPTL4, and ANGPTL8) are also recognized as LPL inhibitors (19). Importantly, these regulatory factors have recently emerged as therapeutic targets with apoCIII and ANGPTL3 antisense oligonucleotides and inhibitory monoclonal antibodies currently being investigated in preclinical and clinical trials (20). Therefore, understanding TRL particle diversity and associated regulatory factors is important to optimally target new therapies in at-risk populations.

The primary aim of the present study was to evaluate lipoprotein abnormalities and markers of TG turnover, undertake nuclear magnetic resonance (NMR) analysis of TRL particle size and distribution, and relate these to the levels of apolipoproteins and angiopoietin-like proteins between healthy controls and patients with SZ.

## Materials and Methods

### Patients

The patients with SZ included in this study were from specialist outpatient clinics at Concord Repatriation General Hospital (Sydney, Australia) monitoring the metabolic and cardiac function of patients with SZ. The healthy controls were recruited from staff and hospital volunteers. The study included subjects over 18 and under 80 years of age, with a clinical diagnosis of SZ receiving antipsychotic medications for more than 1 year. Subjects were excluded if they had major surgery in the last month; a diagnosis of cancer within the last 5 years; active infections, inflammatory disease or were using systemic corticosteroids; liver or renal disease; or were smoking more than 20 cigarettes a day. The study protocol conformed with the ethical guidelines of the 1975 Declaration of Helsinki, and all subjects were recruited with approval from the Sydney Local Health District Human Research Ethics Committee (HREC/11/CRGH/269). Written informed consent was obtained from all subjects. We have previously published on ventricular dysfunction, hypercoagulability, and HDL function in this cohort (21, 22, 23). For the current study, only patients for whom NMR data were available were included, that is, 110 patients and 46 healthy controls. The majority of patients with SZ were prescribed clozapine therapy (91). Non-clozapine patients were receiving olanzapine (8), quetiapine (3), paliperidone (3), risperidone (2), aripiprazole (2), amisulpride (1), or lithium (1), and two patients were prescribed with multiple antipsychotic medications. None of the healthy controls and 21 of the patients with SZ were receiving lipid-lowering therapy (statin).

### Biochemical analyses

Peripheral blood samples were collected from subjects after overnight fasting, from which serum and plasma were isolated. Total cholesterol (TC), HDL-C, and TG were determined using enzymatic tests on a Roche Cobas 8000. LDL-C was calculated using the Friedewald formula (24). Non-HDL-C was calculated by subtracting HDL-C from TC. Remnant cholesterol was calculated by subtracting LDL-C from non-HDL-C. Glycated hemoglobin (HbA1c) was measured by high-pressure liquid chromatography (Biorad). ApoCII (Thermofisher, EHAPOC2), apoCIII (Thermofisher, EHAPOC3), ANGPTL3 (R&D Systems, DY3829), ANGPTL8 (Novus Biologicals, NBP2-68217) and ANGPTL4 (R&D Systems, DY3485) levels were measured in citrate-theophylline-adenosine-dipyridamole (CTAD) plasma by enzyme-linked immunosorbent assay (ELISA). ApoE levels were measured by an in-house designed sandwich ELISA (25). ANGPTL8 and ANGPLT4 levels were measured in 34 and 43 healthy controls and 82 and 106 patients with SZ, respectively. Levels of ANGPTL3 and ANGPTL8 levels were measured in whole plasma, while apoCII, apoCIII, and apoE levels were measured in whole plasma, and in the HDL fraction after precipitation of apoB lipoproteins using polyethylene glycol (PEG). Levels of apoCII, apoCIII, and apoE in the non-HDL fraction were calculated after subtracting HDL fraction values from that of whole plasma.

### PEG precipitation of apoB lipoproteins

CTAD plasma samples were treated with PEG solution (20% PEG in 200 mM glycine buffer, pH 7.4) to precipitate apoB-containing lipoproteins by adding 40 parts of PEG solution to 100 parts plasma. After a 30-min incubation at room temperature, the precipitate was removed by centrifugation (2000 g, 20 min, room temperature) to obtain the PEG supernatant containing the HDL fraction.

### NMR spectroscopy

TRL particle size and distribution were measured by NMR spectroscopy, on a Vantera Clinical Analyzer platform at LabCorp as previously described (26), including analysis of TG and cholesterol content of the various TRL subspecies. Subspecies were defined as: very large TRL particle (VL-TRL-P, 90–240 nm), large TRL particle (L-TRL-P, 50–89 nm), medium TRL particle (M-TRL-P, 37–49 nm), small TRL particle (S-TRL-P 30–36 nm), and very small TRL particle (VS-TRL-P, 24–29 nm). β-hydroxybutyrate, previously reported as a marker of TG turnover per se (11), was also measured by NMR spectroscopy.

### Statistical analysis

Data were reported as frequencies and percentages for categorical variables. Continuous variables were presented as mean ± standard deviation (SD) for normally distributed variables, or median and interquartile range (IQR) for variables not normally distributed. Unadjusted comparisons between healthy controls and patients with SZ were based on unpaired *t* test (parametric distributions), or Mann–Whitney *U* test (nonparametric distributions). General linear modelling analysis was used for comparisons between the two groups after multivariable adjustment for clinical variables including age, sex, body mass index, diabetes, smoking and statin use, with Bonferroni adjustment for multiple comparisons. Variables that were not normally distributed were transformed using log base 10 (log_10_) where appropriate. For zero values, a constant value of 0.01 was added, followed by log_10_ transformation (27). Spearman’s correlation was used to assess univariate associations between variables. *P* values of less than 0.05 in two-tailed testing were considered statistically significant. Analyses were performed using GraphPad Prism 9, or IBM SPSS Statistics (version 28; SPSS Inc).

## Results

### Patients with SZ exhibit elevated TG and remnant cholesterol, without elevation of apoB or β-hydroxybutyrate

The clinical characteristics of healthy controls and patients with SZ in this study are presented in Table 1. In unadjusted analysis, patients with SZ had significantly higher plasma TG levels, significantly lower HDL-C and apoA-I levels, and comparable TC and LDL-C levels to controls (Table 2). Patients with SZ also had significantly higher non-HDL-C and remnant cholesterol levels. Plasma apoB levels were, unexpectedly, similar in controls and patients with SZ (Table 2). There was markedly increased TG:apoB ratio (healthy control median [IQR] 1.0 [0.8–1.7] vs. SZ 2.1 [1.5–2.9], *P* < 0.001), but similar TC:apoB ratio (healthy control 6.0 [4.9–7.1] vs. SZ 5.8 [5.2–6.4], *P* = 0.59) in SZ compared to healthy controls. Plasma β-hydroxybutyrate levels were not different between healthy controls and patients with SZ (Table 2). There were no differences in any of the parameters between patients with SZ receiving clozapine, or not receiving clozapine (data not shown). Multivariable analysis adjusting for clinical characteristics (age, sex, body mass index, diabetes, smoking, and statin use) that might explain the differences between healthy controls and patients with SZ was undertaken (Table 2). Plasma TG levels and TG:apoB ratio remained significantly higher, while HDL-C and apoA-I levels remained significantly lower after multivariable adjustment in patients with SZ in contrast to controls.

**Table 1:** Clinical characteristics of the study cohort (n = 156)

	Healthy Controls (n = 46)	SZ (n = 110)	*P* value
SZ duration of illness, years	-	15.7 (8.6–24.6)	-
Clozapine therapy, %	-	91/110 (83)	-
Age, years	37.5 (30.8–49.0)	41.5 (31.0–52.3)	ns
Biological sex, M/F; %M	22/24 (48)	76/34 (69)	0.01
BMI, kg/m^2^	24.3 (22.9–26.5)	28.5 (25.1–32.8)	<0.001
HbA1c, %	5.4 (5.2–5.5)	5.7 (5.4–6.1)	0.03
Diabetes, %	1/46 (2)	23/110 (21)	0.003
Smoking, % current	0/46 (0)	44/110 (40)	<0.001
Statin therapy, %	0/46 (0)	21/110 (19)	<0.001

Results are expressed as number and (%), or median and (interquartile range). HbA1c levels were determined in 14 controls and 106 patients.

BMI, body mass index; HbA1c, glycated hemoglobin; ns, non-significant; SZ, schizophrenia.

**Table 2:** Unadjusted and multivariable-adjusted analysis of lipid parameters between healthy controls (n = 46) and patient with SZ (n = 110)

	Healthy controls (n = 46)	SZ (n = 110)	*P* value
Unadjusted			
TC, mmol/L	5.0 ± 0.9	5.0 ± 1.0	ns
LDL-C, mmol/L	2.8 ± 0.8	3.0 ± 0.9	ns
HDL-C, mmol/L	1.5 (1.2–1.8)	1.1 (0.9–1.4)	<0.001
TG, mmol/L	0.9 (0.7–1.2)	1.7 (1.2–2.4)	<0.001
Non-HDL-C, mmol/L	3.3 (2.7–4.1)	3.7 (3.0–4.4)	0.02
Remnant cholesterol, mmol/L	0.4 (0.3–0.5)	0.7 (0.5–1.1)	<0.001
apoB, μmol/L	1.7 ± 0.5	1.7 ± 0.4	ns
apoA-I, μmol/L	56.7 (50.0–60.4)	48.6 (42.3–55.4)	<0.001
TG/apoB ratio	1.0 (0.8–1.7)	2.1 (1.5–2.9)	<0.001
TC/apoB ratio	6.0 (4.9–7.1)	5.8 (5.2–6.4)	ns
β-hydroxybutyrate, μmol/L	101.8 (68.0–144.2)	107.3 (77.2–162.0)	ns
Multivariable-adjusted			
TC, mmol/L	4.40 ± 0.22	4.50 ± 0.13	ns
LDL-C, mmol/L	2.13 ± 0.20	2.38 ± 0.12	ns
Log_10_ HDL-C, mmol/L	0.09 ± 0.03	0.04 ± 0.02	0.03
Log_10_ TG, mmol/L	0.11 ± 0.06	0.25 ± 0.03	0.006
Log_10_ Non-HDL-C, mmol/L	0.47 ± 0.03	0.51 ± 0.02	ns
Log_10_ remnant cholesterol, mmol/L	−0.21 ± 0.06	−0.13 ± 0.04	ns
apoB, μmol/L	1.53 ± 0.10	1.49 ± 0.06	ns
Log_10_ apoA-I, μmol/L	1.73 ± 0.02	1.69 ± 0.01	0.005
Log_10_ TG/apoB ratio	0.24 ± 0.05	0.38 ± 0.03	0.002
Log_10_ TC/apoB ratio	0.77 ± 0.02	0.78 ± 0.01	ns
Log_10_ β-hydroxybutyrate, μmol/L	2.05 ± 0.06	2.08 ± 0.04	ns

For unadjusted analysis, results are expressed as mean ± standard deviation, or median and (interquartile range). For multivariable-adjusted analysis, results are expressed as estimated marginal means ± standard error, and non-parametrically distributed variables were log_10_ transformed before analysis. Multivariable model was adjusted for age, sex, body mass index, diabetes, smoking and statin use.

apo, apolipoprotein; HDL-C, high-density lipoprotein cholesterol; LDL-C, low-density lipoprotein cholesterol; log_10_, logarithm base 10; ns, non-significant; SZ, schizophrenia; TC, total cholesterol; TG, triglyceride.

### Patients with SZ have increased concentrations of L-TRL-P and M-TRL-P with similar per particle cholesterol:TG ratios

TRL particle size, concentration (number of particles), and composition (TG and cholesterol content) were determined using NMR. Patients with SZ had evidence of both increased TRL particle size and number (Table 3).

**Table 3:** Unadjusted and multivariable-adjusted analysis of TRL particle size and concentration between healthy controls (n = 46) and patients with SZ (n = 110)

	Healthy Controls (n = 46)	SZ (n = 110)	*P* value
Unadjusted			
Mean TRL-P size, nm	41.0 (37.6–49.2)	54.5 (46.5–61.8)	<0.001
Total TRL-P, nmol/L	113.6 (76.5–201.2)	167.0 (126.2–212.5)	0.002
VL-TRL-P (90–240 nm), nmol/L	0.1 (0–0.1)	0.1 (0–0.8)	ns
L-TRL-P (50–89 nm), nmol/L	0.6 (0–3.0)	7.6 (3.1–13.3)	<0.001
M-TRL-P (37–49 nm), nmol/L	9.4 (4.3–16.2)	24.1 (15.7–37.6)	<0.001
S-TRL-P (30–36 nm), nmol/L	34.9 (20.2–56.5)	26.1 (8.6–47.8)	ns
VS-TRL-P (24–29 nm), nmol/L	60.1 (25.4–132.9)	94.3 (59.9–133.6)	0.02
Multivariable-adjusted			
Log_10_ Mean TRL-P size, nm	1.68 ± 0.02	1.74 ± 0.01	0.001
Log_10_ Total TRL-P, nmol/L	2.03 ± 0.05	2.14 ± 0.03	0.03
Log_10_ VL-TRL-P, nmol/L	−0.82 ± 0.22	−0.91 ± 0.13	ns
Log_10_ L-TRL-P, nmol/L	−0.27 ± 0.22	0.61 ± 0.13	<0.001
Log_10_ M-TRL-P, nmol/L	0.94 ± 0.09	1.31 ± 0.05	<0.001
Log_10_ S-TRL-P, nmol/L	0.66 ± 0.24	0.73 ± 0.14	ns
Log_10_ VS-TRL-P, nmol/L	1.65 ± 0.19	1.81 ± 0.11	ns

All parameters were determined by nuclear magnetic resonance as described in the Materials and methods section. For unadjusted analysis, results are expressed as median and (interquartile range). For multivariable-adjusted analysis, results are expressed as estimated marginal means ± standard error, and non-parametrically distributed variables were log_10_ transformed before analysis. Multivariable model was adjusted for age, sex, body mass index, diabetes, smoking and statin use.

log_10_, logarithm base 10; L-TRL-P, large TRL-P; M-TRL-P, medium TRL-P; ns, non-significant; S-TRL-P, small TRL-P; SZ, schizophrenia; TRL-P, triglyceride-rich lipoprotein particle; VL-TRL-P, very large TRL-P; VS-TRL-P, very small TRL-P.

The most notable differences in particle concentrations were in L-TRL-P (healthy control 0.6 [0–3.0] vs. SZ 7.6 [3.1–13.3] nmol/L, *P* < 0.001) and M-TRL-P (healthy control 9.4 [4.3–16.2] vs. SZ 24.1 [15.7–37.6] nmol/L, *P* < 0.001) where there were 12- and 2.5-fold differences respectively, in unadjusted analysis. More modest differences were seen in VS-TRL-P and S-TRL-P (Table 3). These changes between controls and patients were consistent after multivariable adjustment for clinical characteristics (Table 3). The concentrations of both TG and cholesterol in L-TRL-P, M-TRL-P and VS-TRL-P populations were significantly higher in SZ, however the cholesterol to TG ratios were similar between healthy controls and patients with SZ for all TRL particle populations (Supplemental Table S1).

Various parameters including plasma apoB, TG, non-HDL-C, and remnant cholesterol have been proposed as superior markers for cardiovascular risk beyond LDL-C (28). We, therefore, investigated how these parameters related to TRL particle size and the concentration of various TRL particles in patients with SZ (Supplemental Table S2). In patients with SZ, mean TRL particle size (nm) correlated most strongly with plasma TG and remnant cholesterol (Spearman’s ρ = 0.69 and 0.66, both *P* < 0.001), more weakly with non-HDL-C (ρ = 0.28, *P* < 0.003), and did not correlate with apoB levels. In contrast, total TRL particle concentration (nmol/L) correlated most strongly with plasma apoB and non-HDL-C (ρ = 0.63 and 0.59, both *P* < 0.001) and more weakly but significantly with TG and remnant cholesterol levels (both ρ = 0.36, *P* < 0.001). Plasma apoB, TG, non-HDL-C and remnant cholesterol correlated most strongly with L-TRL-P and M-TRL-P concentrations, and more weakly with VS-TRL-P concentrations (Supplemental Table S2).

### Patients with SZ have higher levels of non-HDL-apoCII, non-HDL-apoCIII, and HDL-apoE

**Fig. 1:**
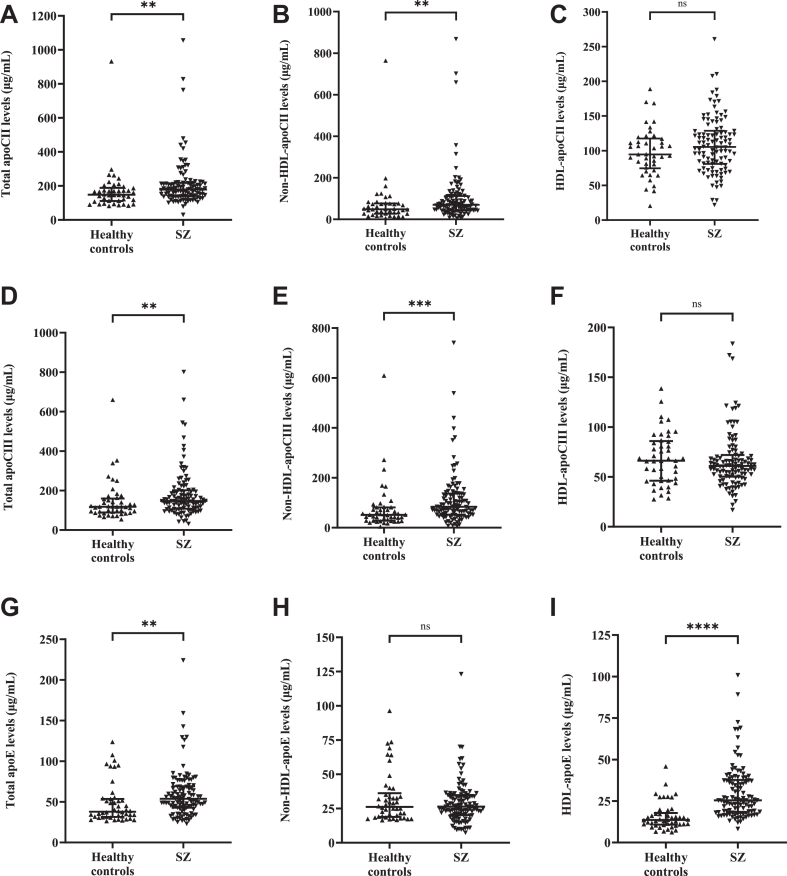
Increased levels of non-HDL-apoCII and apoCIII, and HDL-apoE in patients with SZ. Unadjusted analysis of total (A, D, G), non-HDL (B, E, H), and HDL (C, F, I) levels of apoCII (A–C), apoCIII (D–F) and apoE (G–I) in healthy controls (n = 46) and patients with SZ (n = 110) measured by enzyme-linked immunosorbent assay as described in the Materials and methods. Non-HDL values were determined by subtracting HDL values from total values. Data are median and (interquartile range). ns = non-significant; ∗∗*P* < 0.01; ∗∗∗*P* < 0.001; ∗∗∗∗*P* < 0.0001 versus controls by Mann-Whitney U test. apo, apolipoprotein; HDL, high-density lipoprotein; SZ, schizophrenia.

Apolipoproteins CII, CIII, and E and angiopoietin-like proteins 3, 4, and 8 are important regulators of TG metabolism (14, 16, 19), with apoCII reportedly showing a biphasic relationship to TG levels (29). We therefore measured the circulating levels of these factors in whole plasma, and further separately quantified apoCII, apoCIII, and apoE levels in the HDL and non-HDL fractions as described in Materials and methods. In unadjusted analysis, patients with SZ had significantly increased total and non-HDL, but not HDL-associated apoCII levels (Fig. 1A–C) and apoCIII levels (Fig. 1D–F) as compared to healthy controls. In contrast, total and HDL-associated apoE levels were significantly increased, while non-HDL-apoE levels were not different in patients with SZ (Fig. 1G–I). Circulating ANGPTL3, ANGPTL4, and ANGPTL8 levels were not different between healthy controls and patients with SZ (Supplemental Fig. S1). In multivariable-adjusted analysis, non-HDL-apoCIII and HDL-apoE levels remained significantly increased in patients with SZ compared to controls (Supplemental Table S3), indicating that elevated apoCIII and apoE levels were incompletely explained by conventional clinical risk factors.

### Plasma TG, remnant cholesterol, and large and medium TRL particles positively correlate with apoCII, apoCIII, and apoE levels in patients with SZ

ApoCII, apoCIII, apoE, ANGPTL3, ANGPTL4, and ANGPTL8 are all major regulators of TRL lipolysis and clearance (14, 19). As such, we examined how these regulatory factors are associated with TG parameters and TRL particle concentrations in patients with SZ. ApoB levels positively correlated with HDL-associated apoCII and apoCIII levels, and non-HDL and HDL-associated apoE levels (Table 4). TG, non-HDL-C and remnant cholesterol levels all positively correlated with non-HDL levels of apoCII, and non-HDL and HDL levels of apoCIII and apoE (Table 4). ANGPTL3 levels weakly and negatively correlated with TG and remnant cholesterol levels, while ANGPTL4 and ANGPTL8 levels did not correlate with apoB, TG, non-HDL-C or remnant cholesterol levels (Table 4). Correlations between apoCII, apoCIII, and apoE levels and individual TRL particle population concentrations are summarized in Table 5. They confirm a highly significant positive correlation between non-HDL and HDL-apoCII, apoCIII, and apoE levels with L-TRL-P and M-TRL-P concentrations in patients with SZ. Non-HDL-apoCIII levels also positively correlated with VL-TRL-P concentrations, while non-HDL-apoCII levels negatively correlated with S-TRL-P concentrations (Table 5). HDL-apoCII, apoCIII and apoE levels all positively correlated with VS-TRL-P concentrations (Table 5). ANGPTL3, ANGPTL4, and ANGPTL8 levels did not correlate with any of the TRL particles (data not shown).

**Table 4:** Spearman’s rank correlation coefficients between apoCII, apoCIII, apoE, ANGPTL3, ANGPTL4 and ANGPTL8 levels and apoB, TG, non-HDL-C, and remnant cholesterol levels in patients with schizophrenia

	apoB, μmol/L		TG, Mmol/L		Non-HDL-C, Mmol/L		Remnant Cholesterol, Mmol/L	
	ρ	*P* value	ρ	*P* value	ρ	*P* value	ρ	*P* value
apoCII, μg/ml								
Total apoCII	0.24	0.01	0.44	<0.001	0.33	<0.001	0.40	<0.001
Non-HDL-apoCII	0.10	ns	0.50	<0.001	0.23	0.007	0.46	<0.001
HDL-apoCII	0.26	0.009	0.18	ns	0.26	0.02	0.13	ns
apoCIII, μg/ml								
Total-apoCIII	0.12	ns	0.38	<0.001	0.29	0.003	0.34	<0.001
Non-HDL-apoCIII	0.07	ns	0.35	<0.001	0.25	0.009	0.30	0.002
HDL-apoCIII	0.26	0.007	0.36	<0.001	0.33	<0.001	0.31	0.001
apoE, μg/ml								
Total-apoE	0.36	<0.001	0.41	<0.001	0.41	<0.001	0.37	<0.001
Non-HDL-apoE	0.27	0.005	0.27	0.005	0.31	<0.001	0.24	0.01
HDL-apoE	0.33	<0.001	0.37	<0.001	0.37	<0.001	0.33	<0.001
ANGPTL								
ANGPTL3, ng/ml	0.004	ns	−0.21	0.03	−0.03	ns	−0.22	0.023
ANGPTL4, ng/ml	0.14	ns	0.06	ns	0.04	ns	0.003	ns
ANGPTL8, pg/ml	−0.200	ns	−0.05	ns	−0.13	ns	−0.05	ns

ANGPTL, angiopoietin-like protein; apo, apolipoprotein; HDL, high-density lipoprotein; HDL-C, high-density lipoprotein cholesterol; ns, non-significant; TG, triglyceride.

**Table 5:** Spearman’s rank correlation coefficients between apoCII, apoCIII and apoE levels and TRL-P concentrations in patients with schizophrenia

	Total TRL-P, Nmol/L		VL-TRL-P, Nmol/L		L-TRL-P, Nmol/L		M-TRL-P, Nmol/L		S-TRL-P, Nmol/L		VS-TRL-P, Nmol/L	
	ρ	*P* value	ρ	*P* value	ρ	*P* value	ρ	*P* value	ρ	*P* value	ρ	*P* value
apoCII, μg/ml												
Total apoCII	0.34	<0.001	0.170	ns	0.53	<0.001	0.45	<0.001	−0.12	ns	0.26	0.007
Non-HDL-apoCII	0.17	ns	0.19	ns	0.56	<0.001	0.40	<0.001	−0.30	0.002	0.18	ns
HDL-apoCII	0.36	<0.001	0.06	ns	0.20	0.047	0.29	0.003	0.12	ns	0.22	0.03
apoCIII, μg/ml												
Total-apoCIII	0.25	0.011	0.26	0.007	0.46	<0.001	0.32	<0.001	−0.07	ns	0.17	ns
Non-HDL-apoCIII	0.15	ns	0.27	0.005	0.40	<0.001	0.24	0.01	−0.11	ns	0.09	ns
HDL-apoCIII	0.38	0.001	0.18	ns	0.40	<0.001	0.38	<0.001	0.01	ns	0.30	0.002
apoE, μg/ml												
Total-apoE	0.47	<0.001	0.20	0.04	0.54	<0.001	0.55	<0.001	−0.04	ns	0.36	<0.001
Non-HDL-apoE	0.34	<0.001	0.16	ns	0.41	<0.001	0.38	<0.001	0.03	ns	0.29	0.002
HDL-apoE	0.42	<0.001	0.23	0.02	0.48	<0.001	0.50	<0.001	−0.04	ns	0.28	0.003

apo, apolipoprotein; HDL, high-density lipoprotein; L-TRL-P, large TRL-P; M-TRL-P, medium TRL-P; ns, non-significant; S-TRL-P, small TRL-P; TRL-P, triglyceride-rich lipoprotein particle; VL-TRL-P, very large TRL-P; VS-TRL-P, very small TRL-P.

## Discussion

Overwhelming evidence from genetic, epidemiological, preclinical, and clinical studies provides strong support for hypertriglyceridemia as a causal risk factor for atherosclerotic cardiovascular disease (ASCVD). Rather than a uniform disorder, hypertriglyceridemia should be considered as a spectrum of metabolic alterations in which the assessment of associated cardiovascular risk, and implementation of appropriate intervention strategies, requires a context-specific understanding of the key mechanistic pathways and factors involved. In this study, we found that patients with SZ demonstrated elevated TG, non-HDL-C, and remnant cholesterol levels all of which are important risk factors for ASCVD (28). They demonstrated similar LDL-C and apoB levels to healthy controls and therefore had increased TG to apoB ratios. β-hydroxybutyrate, a ketone body derived from free fatty acids that was recently reported as a marker of TG turnover *per* se (11), was not increased in patients with SZ as compared to healthy controls. NMR analysis revealed that patients with SZ had more TRL particles, with a marked increase in large (50–89 nm) and medium-sized (37–49 nm) particle concentrations, which were compositionally similar to those of healthy controls with respect to cholesterol to TG ratio. Large and medium TRL particle numbers positively correlated with elevated levels of apoCII and apoCIII and apoE. As these marked changes were incompletely explained by clinical characteristics and comorbidities such as obesity (BMI), diabetes and smoking, this suggests a severe TG-enriched phenotype insufficiently explained by conventional risk factors in SZ.

Patients with SZ had elevated remnant cholesterol levels. Elevated remnant cholesterol is associated with a pro-atherogenic profile that is linked to residual risk of ASCVD (30, 31, 32). Remnant particles can carry up to four times more cholesterol content per particle than LDLs and may therefore potentially be much more atherogenic (7). Mechanistic studies show that like LDL-C, remnant cholesterol can also directly mediate atherogenesis by promoting inflammation, endothelial dysfunction, foam cell generation and pro-thrombotic events (9, 33). Recently, Wadström and colleagues reported that elevated remnant cholesterol and low-grade inflammation explained 24% and 18% of the excess risk of any ASCVD event, respectively, in individuals with diabetes from the Copenhagen General Population Study (34). It would be interesting in future studies to confirm elevation of calculated remnant cholesterol using direct measurement of defined remnant particles (35) which may provide a more precise measure of the remnant population.

In patients with abdominal obesity and diabetes, insulin resistance and excess dietary intake are positively associated with hepatic TRL production, and therefore are major determinants of plasma TG levels (36, 37). The increase in the concentrations of large and medium TRL particles in patients with SZ, however, also indicates impaired TRL lipolysis and clearance, which has been shown to be the predominant regulator of large and medium particles in the VLDL_1_ (50–80 nm) and VLDL_2_ (30–50 nm) size ranges (38). As such, elevated TG and remnant cholesterol in patients with SZ may be due to the combination of both increased production, and reduced lipolysis and clearance of TRL and remnant particles. The observation that β-hydroxybutyrate levels were not significantly different in patients with SZ should be interpreted cautiously as β-hydroxybutyrate is a marker for fatty acid oxidation that can relate to a diverse range of other metabolic processes, and therefore does not exclusively reflect TG turnover. Further examination of other turnover markers is required to confirm if TG turnover is altered in SZ.

The link between antipsychotic medications and hypertriglyceridemia is well described. Many agents are orexigenic and are associated with weight gain, obesity, and dyslipidemia (39, 40, 41), and there may be a genetic susceptibility to antipsychotic induced hypertriglyceridemia in schizophrenia (42). However, there is also evidence of more prevalent metabolic syndrome and dyslipidemia in naïve, untreated patients (43, 44). It is worth noting that in our study, TG, TRL particle concentrations, and apoCIII remained elevated in patients with SZ even after adjusting for BMI, suggesting that these effects may be independent of antipsychotic-induced weight gain. In our study, patient numbers were too small to investigate for differences between antipsychotics, and, as our study only included patients who were stable on long-term treatment with antipsychotics a non-treated control group was not feasible and this remains a study limitation.

Apolipoproteins play important roles in regulating TRL clearance and removal, and because they actively exchange between lipid surfaces, they can be found on both TRLs and HDLs. While the majority of studies have reported on total circulating levels, we have reported levels in both the HDL and non-HDL (representing TRL) fractions of whole plasma to capture the levels found in the different lipoprotein populations, where they serve different functions. TRL-associated apoCII and apoCIII are involved in regulating LPL activity, TRL lipolysis, and clearance (14), whereas the function of apoCII and apoCIII on HDLs remains unclear. There is some evidence suggesting that HDL-containing apoCIII is associated with reduced HDL anti-atherogenic properties and higher cardiovascular risk (45, 46). In this study, patients with SZ had significantly elevated levels of apoCII and apoCIII in whole plasma and the non-HDL fraction, whereas levels in the HDL fraction were not significantly different compared to healthy controls. The observation that HDL-apoCII and -apoCIII are positively associated with concentrations of large and medium TRL particles may reflect the dynamic exchange of apoCII and apoCIII pools between TRLs and HDLs (47).

ApoCIII is an established inhibitor of LPL activity (14) and receptor-mediated uptake of TRL and remnant particles through the liver (17, 18). The increased non-HDL-apoCIII in association with large and medium TRL particles supports the idea that TRL lipolysis and clearance are impaired in SZ, and is consistent with Boiko *et al.* who showed that serum apoCIII levels were increased in patients with SZ in contrast to healthy individuals, and more pronounced in patients with metabolic syndrome (48). Similar findings have also been shown in patients with metabolic syndrome and type 2 diabetes mellitus without severe mental illness (49, 50). Genetic, Mendelian randomization and large-scale population studies strongly support the causative role of apoCIII in increasing plasma TG and the risk of ASCVD (51, 52). In one meta-analysis study, lower risk of ischaemic vascular disease associated with *APOC3* loss-of-function was mediated by lower remnant cholesterol, but not LDL-C (53). As such, novel molecules targeting apoCIII currently under investigation in preclinical and clinical trials are promising therapeutic candidates for the treatment of moderate hypertriglyceridemia to prevent ASCVD (54) and may be applicable to patients with SZ given their elevated TG, TRL concentration, and non-HDL-apoCIII levels.

ApoCII is an essential cofactor for LPL, and while apoCII deficiency is associated with reduced LPL activity and marked hypertriglyceridemia (14), excess levels have also been inconsistently associated with elevated TG levels (55, 56). It has been proposed that excess apoCII may interfere with the physical access of LPL to TRLs, and that optimal levels are required for proper enzyme-to-substrate interaction and efficient lipolysis (57). Consistent with these previous observations, a recent study in coronary artery disease patients reported a bell-shaped effect of apoCII concentrations on glycosylphosphatidylinositol high-density-lipoprotein binding protein-1 associated LPL activity, in which extremely high concentrations of apoCII were associated with reduced LPL activity levels comparable to that observed in the absence of apoCII (29). Additionally, excess apoCII has also been shown to inhibit apoB/apoE-mediated binding of TRLs to the LDL receptor (LDLR) or LDLR-related protein 1 (LRP-1), indicating that elevated apoCII levels may also impede hepatic uptake and removal of TRL particles (17, 18). As the levels of non-HDL-associated apoCII in patients with SZ were in excess of optimal levels reported (58, 59), and were also positively associated with large and medium TRL particle concentrations, elevated apoCII may be inhibiting TRL lipolysis and clearance in SZ. Thus, while lowering apoCIII with emerging biological therapies remains a feasible strategy in SZ, raising apoCII to enhance LPL activity does not appear plausible in these patients.

ApoE facilitates the binding of lipoproteins to hepatic receptors such as the LDLR and is therefore a crucial factor in regulating lipoprotein uptake and removal. Higher levels of circulating apoE are found in populations with increased risk of cardiovascular disease, although in many cases it is not clear whether this is contributed to by apoE in HDL, or in non-HDL fractions (60). In our study, HDL-associated apoE was increased, while non-HDL-associated apoE was not different, suggesting that apoE-dependent TRL and remnant hepatic uptake and clearance is unlikely to be impaired in SZ. ApoE on HDLs may facilitate HDL removal and clearance, and also augment its anti-atherogenic properties by promoting ATP-binding cassette transporter A1 (ABCA1) and ATP-binding cassette transporter G1 (ABCG1)-dependent cholesterol efflux (61, 62, 63). In obese and coronary artery disease patients, high levels of HDL containing apoE but lacking apoCIII are associated with reduced coronary plaque burden and cardiovascular risk, whereas HDL containing both apoE and apoCIII are associated with increased disease burden and cardiovascular risk (64, 65). Further studies clarifying HDL particle apolipoprotein content in patients with SZ are needed.

ANGPTL3 and ANGPTL4 are well-known inhibitors of LPL and TRL lipolysis, and loss of function mutations in the *ANGPTL3* and *ANGPTL4* genes are associated with increased LPL activity and reduced TG levels (19, 66). Importantly, the functions of both ANGPTL3 and ANGPTL4 are modulated by their association with ANGPTL8. ANGPTL3 function is augmented while that of ANGPLT4 is antagonized when complexed with ANGPTL8 to enhance and suppress inhibition of LPL activity respectively (19, 67). ANGPTL3 and ANGPLT4 can also inhibit endothelial and/or hepatic lipase activity, which are involved in the clearance of TRL remnants (68, 69). Furthermore, ANGPTL3 may be directly involved in the hepatic uptake of TRL remnants (70). We found that the levels of ANGPTL3, ANGPTL4, and ANGPTL8 were not significantly different between healthy controls and patients with SZ, and did not correlate with TG, non-HDL-C, remnant cholesterol, or TRL levels. Similar findings have been reported in other populations, and few associations were found between ANGPTL3, ANGPTL4, or ANGPLT8 levels with features of metabolic syndrome (71, 72, 73, 74). On the other hand, studies have also reported increased circulating levels of ANGPTL3, ANGPTL4 or ANGPTL8 in association with metabolic syndrome in cohorts with obesity, diabetes and/or dyslipidemia (75, 76). The inconsistent results in previous studies, and negative results in the present study suggest that levels of ANGPTL3, ANGPTL4 and ANGPTL8 are not helpful as markers, although these regulatory factors may still remain candidate targets for inhibition in SZ given likely impaired lipolytic activity.

In conclusion, we have demonstrated for the first time that in SZ there is increased plasma TG, non-HDL-C, and remnant cholesterol but very similar apoB levels to healthy individuals. The increased TG and remnant cholesterol is expected to be pro-atherogenic and is associated with marked elevation of large and medium TRL particles consistent with impaired lipolysis. Future therapies enhancing TRL lipolysis and clearance appear plausible targets for these patients.

## Data Availability

The data in this article are presented in the tables, figures or supplemental data. Dr Jeffrey Wang had full access to all the data in the study and takes responsibility for its integrity and the data analysis. Additional data are available upon reasonable request from the corresponding author L.K. (Atherosclerosis Laboratory, The ANZAC Research Institute, Concord Repatriation General Hospital and the University of Sydney, Sydney, Australia; leonard.kritharides@sydney.edu.au).

## Supplemental data

This article contains supplemental data.

## Conflict of interest

The authors declare the following financial interests/personal relationships which may be considered as potential competing interests:

L.K. has participated in clinical trials sponsored by Amgen and Novartis; he has given lectures and received consulting fees from Seqiris (CSL), Amgen and Novartis. D.S. has participated in clinical trials sponsored by NHMRC Clinical Trials Centre; he has received support from Arrowhead Pharmaceuticals for previous publications; he has received grants/contracts sponsored by Regeneron Pharmaceuticals, Ionis Pharmaceuticals, Arrowhead Pharmaceuticals, Amgen and Novartis; he has given lectures and received consulting fees from Amgen and Novartis; he has received contributions from Sanofi.
