# Supplementary material for: Triglyceride-rich lipoprotein, remnant cholesterol, and apolipoproteins CII, CIII, and E in patients with schizophrenia
Source: J Lipid Res. 2024 Jun 13;65(7):100577. doi: 10.1016/j.jlr.2024.100577 (PMC11304881; doi:10.1016/j.jlr.2024.100577)
Supplement: Supplemental Data [file mmc1.docx]

**Supplemental Data**

|  | **Healthy controls  (n = 46)** | **SZ  (n = 110)** | ***p* value** |
| --- | --- | --- | --- |
| **TG, mmol/L** | | | |
| VL-TRL-P (90-240 nm) | 0.08 (0.05-0.12) | 0.09 (0.04-0.34) | ns |
| L-TRL-P (50-89 nm) | 0.03 (0-0.19) | 0.56 (0.20-0.98) | <0.001 |
| M-TRL-P (37-49 nm) | 0.17 (0.09-0.32) | 0.48 (0.27-0.69) | <0.001 |
| S-TRL-P (30-36 nm) | 0.15 (0.08-0.22) | 0.10 (0.03-0.15) | 0.008 |
| VS-TRL-P (24-29 nm) | 0.09 (0.03-0.17) | 0.14 (0.09-0.20) | 0.009 |
| **Cholesterol, mmol/L** | | | |
| VL-TRL-P | 0.01 (0.01-0.02) | 0.01 (0-0.06) | ns |
| L-TRL-P | 0.01 (0-0.07) | 0.19 (0.07-0.34) | <0.001 |
| M-TRL-P | 0.07 (0.04-0.13) | 0.20 (0.11-0.30) | <0.001 |
| S-TRL-P | 0.15 (0.10-0.21) | 0.11 (0.04-0.18) | 0.03 |
| VS-TRL-P | 0.20 (0.08-0.39) | 0.32 (0.20-0.46) | 0.009 |
| **Cholesterol:TG ratio** | | | |
| VL-TRL-P | 0.14 (0.10-0.17) | 0.13 (0.08-0.21) | ns |
| L-TRL-P | 0.35 (0-0.36) | 0.36 (0.33-0.36) | ns |
| M-TRL-P | 0.41 (0.40-0.42) | 0.41 (0.40-0.43) | 0.04 |
| S-TRL-P | 1.09 (0.99-1.11) | 1.11 (1.02-1.35) | ns |
| VS-TRL-P | 2.29 (2.26-2.29) | 2.27 (2.26-2.29) | ns |
| All parameters were determined by nuclear magnetic resonance as described in the methods section. Results are expressed as mean ± standard deviation, or median and (interquartile range).  log_10_ = logarithm base 10; ns = non-significant; SZ = schizophrenia; TG = triglyceride;  TRL-P = triglyceride-rich lipoprotein particle; VL-TRL-P = very large TRL-P;  L-TRL-P = large TRL-P; M-TRL-P = medium TRL-P; S-TRL-P = small TRL-P;  VS-TRL-P = very small TRL-P. | | | |

**Supplemental Table S1: Analysis of TRL particle TG and cholesterol content between healthy controls (n=46) and patients with SZ (n=110).**

**Supplemental Table S2: Spearman’s rank correlation coefficients between TRL-P size and number and apoB, TG, non-HDL-C, and remnant cholesterol levels in patients with schizophrenia.**

|  | **apoB, µmol/L** | | **TG, mmol/L** | | **Non-HDL-C, mmol/L** | | **Remnant cholesterol, mmol/L** | |
| --- | --- | --- | --- | --- | --- | --- | --- | --- |
|  | **ρ** | ***p* value** | **ρ** | ***p* value** | **ρ** | ***p* value** | **ρ** | ***p* value** |
| Mean TRL-P size, nm | 0.12 | ns | 0.69 | <0.001 | 0.28 | 0.003 | 0.66 | <0.001 |
| Total TRL-P number, nmol/L | 0.63 | <0.001 | 0.36 | <0.001 | 0.59 | <0.001 | 0.36 | <0.001 |
| VL-TRL-P number, nmol/L | 0.03 | ns | 0.46 | <0.001 | 0.18 | ns | 0.41 | <0.001 |
| L-TRL-P number, nmol/L | 0.39 | <0.001 | 0.75 | <0.001 | 0.47 | <0.001 | 0.74 | <0.001 |
| M-TRL-P number, nmol/L | 0.42 | <0.001 | 0.55 | <0.001 | 0.47 | <0.001 | 0.56 | <0.001 |
| S-TRL-P number, nmol/L | 0.26 | 0.007 | -0.17 | ns | 0.16 | ns | -0.15 | ns |
| VS-TRL-P number, nmol/L | 0.37 | <0.001 | 0.27 | 0.004 | 0.34 | <0.001 | 0.25 | 0.009 |
| ns = non-significant; apo = apolipoprotein; TG = triglyceride; HDL-C = high-density lipoprotein cholesterol; TRL-P = triglyceride-rich lipoprotein particle; VL-TRL-P = very large TRL-P;  L-TRL-P = large TRL-P; M-TRL-P = medium TRL-P; S-TRL-P = small TRL-P; VS-TRL-P = very small TRL-P. | | | | | | | | |

|  | **Healthy controls  (n = 46)** | **SZ  (n = 110)** | ***p* value** |
| --- | --- | --- | --- |
| **apoCII, µg/mL** | | | |
| Log_10_ Total apoCII | 2.20 ± 0.05 | 2.27 ± 0.03 | ns |
| Log_10_ Non-HDL-apoCII | 1.75 ± 0.09 | 1.88 ± 0.06 | ns |
| Log_10_ HDL-apoCII | 1.96 ± 0.05 | 1.99 ± 0.03 | ns |
| **apoCIII, µg/mL** | | | |
| Log_10_ Total apoCIII | 2.10 ± 0.06 | 2.18 ± 0.03 | ns |
| Log_10_ Non-HDL-apoCIII | 1.69 ± 0.09 | 1.88 ± 0.05 | 0.02 |
| Log_10_ HDL-apoCIII | 1.81 ± 0.04 | 1.80 ± 0.02 | ns |
| **apoE, µg/mL** |  |  |  |
| Log_10_ Total apoE | 1.64 ± 0.04 | 1.71 ± 0.03 | ns |
| Log_10_ Non-HDL-apoE | 1.44 ± 0.05 | 1.38 ± 0.03 | ns |
| Log_10_ HDL-apoE | 1.17 ± 0.05 | 1.42 ± 0.03 | <0.001 |
| **ANGPTL** |  |  |  |
| Log_10_ ANGPTL3, ng/mL | 2.49 ± 0.03 | 2.49 ± 0.02 | ns |
| Log_10_ ANGPTL4, ng/mL | 1.98 ± 0.12 | 2.05 ± 0.07 | ns |
| Log_10_ ANGPTL8, pg/mL | 1.86 ± 0.10 | 1.84 ± 0.06 | ns |
| Results are expressed as estimated marginal means ± standard error, and non-parametrically distributed variables were log_10_ transformed before analysis. ANGPTL4 analysis was performed in n=43 healthy controls and n=106 patients with SZ whilst ANGPTL8 analysis performed in n=34 healthy controls and n=82 patients with SZ. Multivariable models were adjusted for age, sex, body mass index, diabetes, smoking and statin use. log_10_ = logarithm base 10; ns = non-significant;  SZ = schizophrenia; apo = apolipoprotein; HDL = high-density lipoprotein;  ANGPTL = angiopoietin-like protein. | | | |

**Supplemental Table S3: Multivariable-adjusted analysis of apoCII, apoCIII, apoE, ANGPTL3, ANGPTL4 and ANGPTL8 levels between healthy controls (n=46) and patients with SZ (n=110).**

**(C)**

**(B)**

**(A)**

**Supplemental Figure S1: ANGPTL3, ANGPTL8 and ANGPTL4 levels in healthy controls and patients with SZ.** Unadjusted analysis of ANGPTL3 (A), ANGPTL4 (B) and ANGPTL8 (C) levels in healthy controls and patients with SZ, measured by enzyme-linked immunosorbent assay as described in the methods. Data are median and (interquartile range).
ns = non-significant vs controls by Mann-Whitney U test. SZ = schizophrenia;
ANGPTL = angiopoietin-like protein.
